# The feasibility of a strategy for the remote recruitment, consenting and assessment of recent referrals: a protocol for phase 1 of the On-Line Parent Training for the Initial Management of ADHD referrals (OPTIMA)

**DOI:** 10.1186/s40814-021-00959-0

**Published:** 2022-01-03

**Authors:** Katarzyna Kostyrka-Allchorne, Claire Ballard, Sarah Byford, Samuele Cortese, David Daley, Johnny Downs, Blandine French, Cristine Glazebrook, Kimberley Goldsmith, Madeleine J. Groom, Charlotte L. Hall, Ellen Hedstrom, Zina Ibrahim, Christine Jarvis, Hanna Kovshoff, Jana Kreppner, Nancy Lean, Anna Morris, Walter Muruet Gutierrez, Kapil Sayal, James Shearer, Emily Simonoff, Margaret Thompson, Lukasz Zalewski, Edmund J. S. Sonuga-Barke

**Affiliations:** 1 Department of Child and Adolescent Psychiatry, Institute of Psychiatry, Psychology & Neuroscience, King’s College London, 16 De Crespigny Park, London, SE5 8AF UK; 2 Department of Health Service and Population Research, Institute of Psychiatry, Psychology & Neuroscience, King’s College London, London, UK; 3 Centre for Innovation in Mental Health, School of Psychology, Faculty of Environmental and Life Sciences, University of Southampton, Southampton, UK; 4 Clinical and Experimental Sciences (CNS and Psychiatry), Faculty of Medicine, University of Southampton, Southampton, UK; 5 Solent NHS Trust, Southampton, UK; 6 Hassenfeld Children’s Hospital at NYU Langone, New York University Child Study Center, New York City, New York USA; 7 Academic Unit of Mental Health & Clinical Neurosciences, Faculty of Medicine & Health Sciences, University of Nottingham, Nottingham, UK; 8 Centre for ADHD and Neurodevelopmental Disorders Across the Lifespan CANDAL Institute of Mental Health, University of Nottingham, Nottingham, UK; 9 South London and Maudsley NHS Foundation Trust, London, UK; 10 NIHR MindTech MedTech Co-operative, Institute of Mental Health, University of Nottingham, Nottingham, UK; 11 Department of Biostatistics & Health Informatics, Institute of Psychiatry, Psychology & Neuroscience, King’s College London, London, UK; 12 ADHD Solutions Community Interest Company, Leicester, UK; 13 Department of Child & Adolescent Psychiatry, Aarhus University, Aarhus, Denmark

**Keywords:** ADHD, Conduct problems, Oppositional defiant disorder, Waiting list, Digital intervention, Parent training

## Abstract

**Background:**

In the UK, children with high levels of hyperactivity, impulsivity and inattention referred to clinical services with possible attention-deficit/hyperactivity disorder (ADHD) often wait a long time for specialist diagnostic assessment. Parent training (PT) has the potential to support parents during this difficult period, especially regarding the management of challenging and disruptive behaviours that often accompany ADHD. However, traditional face-to-face PT is costly and difficult to organise in a timely way. We have created a low-cost, easily accessible PT programme delivered via a phone app, Structured E-Parenting Support (STEPS), to address this problem. The overall OPTIMA programme will evaluate the efficacy and cost-effectiveness of STEPS as a way of helping parents manage their children behaviour while on the waitlist. To ensure the timely and efficient evaluation of STEPS in OPTIMA, we have worked with children’s health services to implement a remote strategy for recruitment, screening and assessment of recently referred families. Part of this strategy is incorporated into routine clinical practice and part is OPTIMA specific. Here, we present the protocol for Phase 1 of OPTIMA—a study of the feasibility of this remote strategy, as a basis for a large-scale STEPS randomised controlled trial (RCT).

**Methods:**

This is a single arm observational feasibility study. Participants will be parents of up to 100 children aged 5-11 years with high levels of hyperactivity/impulsivity, inattention and challenging behaviour who are waiting for assessment in one of five UK child and adolescent mental health or behavioural services. Recruitment, consenting and data collection will occur remotely. The primary outcome will be the rate at which the families, who meet inclusion criteria, agree in principle to take part in a full STEPS RCT. Secondary outcomes include acceptability of remote consenting and online data collection procedures; the feasibility of collecting teacher data remotely within the required timeframe, and technical difficulties with completing online questionnaires. All parents in the study will receive access to STEPS.

**Discussion:**

Establishing the feasibility of our remote recruitment, consenting and assessment strategy is a pre-requisite for the full trial of OPTIMA. It can also provide a model for future trials conducted remotely.

**Supplementary Information:**

The online version contains supplementary material available at 10.1186/s40814-021-00959-0.

## Background

Attention-deficit/hyperactivity disorder (ADHD) is a debilitating neurodevelopmental condition that is characterised by symptoms of inattention and/or impulsivity and hyperactivity and affects about 4% of UK children [1]. ADHD is associated with substantial impairment for the child across multiple life domains [2–5]. It also negatively affects carers’ mental health and family wellbeing [6]. Up to 90% of children with an ADHD diagnosis also display a broader pattern of behaviour problems (e.g., oppositional, disruptive, defiant and challenging behaviours) and often meet the criteria for oppositional defiant disorder (ODD) [7]. This exacerbates the levels of impairment experienced by children with ADHD [8] and presents a major challenge to parents, increasing levels of parenting stress [9] and mental health problems [10]. These challenging behaviours contribute to coercive child-parent relationships that can deteriorate over time [11]. It is the escalation of these challenging behaviours to crisis levels that often leads parents with children, who have high levels of hyperactivity/impulsivity and inattention, to first seek professional help [12]. For many parents, finding a way to manage their child’s disruptive and defiant behaviour is likely to be the most urgent treatment priority at the time of their referral to child and adolescent mental health services (CAMHS).

Consistent with this, the National Institute for Health and Care Excellence (NICE) recommends that parent training should be made available to families whose children are referred with hyperactivity/impulsivity and inattention, especially where these are accompanied by challenging and disruptive behaviour, soon after their referral is made, allowing them to start addressing their children’s behavioural difficulties early on [1]. Parent training is an existing evidence-based intervention for disruptive and defiant behaviour displayed by children with ADHD, which helps parents to find more effective ways to manage their children’s behaviour [13].

However, currently, the means to deliver this intervention in a timely manner soon after a referral is lacking. This is because parent training is traditionally delivered face-to-face either in small groups or on a one-to-one basis and is costly to provide and time-consuming to organise [14]. Because of this, typically, parent training is only offered after a full-clinical assessment has been made and an ADHD diagnosis is given. The long waiting lists that currently exist for CAMHS mean that parents can be left without support and guidance for extended periods while waiting for the assessment. For example, the Children’s Commissioner Lightening Report in 2016 found up to 200 days delay to initial assessment [15]. Having to wait for parent training carries a substantial risk of further deterioration of the parent-child relationship and escalation of their child’s problems—with concomitant probable effects on parent and family wellbeing that this implies.

To address this problem, we have developed a new digital way to deliver support to parents to help them manage the challenging behaviour of their children referred with hyperactivity/impulsivity and inattention—Structured E-Parenting Support (STEPS). STEPS is a low-cost, easy and quick to access parenting support intervention delivered as a mobile phone application (app). It is designed to provide established evidence-based advice and support for parents, whose children display hyperactivity/impulsivity, inattention, to help them better manage their children’s co-occurring disruptive and oppositional behaviours. Inspired by the New Forest Parenting Programme [16], a face-to-face parent training programme, its content has been shaped by the latest research about parenting and child behaviour [13, 14, 17] as well as many years of clinical experience and the views of families of children with ADHD-type problems. Key treatment objectives include: (1) increasing parents’ knowledge of why some children’s behaviour is more difficult to manage than others, (2) building their child’s confidence, (3) facilitating effective communication patterns and (4) giving parents strategies to facilitate more effective management of their child’s challenging behaviour. The content is delivered in eight steps, each following a standard structure with common audio-visual and graphic elements describing and illustrating recommended parenting practices and skills. For a more detailed overview of STEPS, see Supplementary Documents (1. Structured E-Parenting Support).

## The present study

We have been funded by the UK National Institute for Health Research (NIHR) to evaluate STEPS as a way of delivering parent training to families of children who have been referred to children’s services and are waiting for specialist assessment and treatment. The programme of research is called *On-Line Parent Training for the Initial Management of ADHD referrals* (OPTIMA [RP-PG-0618-20003]). Its main component is a large multi-centre randomised control trial (RCT) comparing STEPS with waiting as usual (STEPS RCT). However, before we can proceed with this full RCT, we need to establish that we can recruit sufficient new CAMHS and early behavioural services referrals with hyperactivity/impulsivity, inattention and challenging behaviour to provide adequate statistical power for this evaluation. To this end, we have worked with children’s services to implement a remote digital strategy for recruitment, screening and assessment of recently referred families—part of which is incorporated into routine clinical practice and part of which is OPTIMA specific. Using remote digital methods ensures rapid and systematic screening of ADHD and ODD symptoms in children accepted by children’s health services from different referral sources and provide parents with access to support as soon as possible, in the form of STEPS. This strategy also reduces administrative burden and the gatekeeping function of local clinicians in a way that gives parents greater autonomy in decisions to participate in research. The aim of Phase 1 of the OPTIMA programme is to assess the feasibility of this remote strategy as a basis for a full-scale RCT.

## Methods

### Study objective

The primary objective of the study is to test whether we can use our remote recruitment, consenting and assessment strategy to recruit recent referrals to children’s services (CAMHS and early behavioural support services) at a sufficient monthly rate to provide statistical power for our planned STEPS RCT.

Secondary objectives include establishing the usability and acceptability of our remote recruitment, consenting and assessment strategy including the online outcome and adverse events collection procedure. We will also assess the feasibility of collecting teacher data remotely within the required timeframe and capture technical difficulties completing online questionnaires.

This study will not pilot or test the feasibility of the STEPS app as a form of a parent training intervention.

### Design

This will be a single-arm observational feasibility study.

### Setting

The study will be conducted remotely (using phone calls and digital methods), with researchers based in three trial centres: London, Southampton and Nottingham (UK). Recruitment will take place across five sites: South London and Maudsley NHS Trust (SLaM), Solent NHS Trust, North East London NHS Foundation Trust (NELFT), Black Country Healthcare NHS Foundation Trust and Nottingham City Council. These five sites have been selected as they have (i) an early triage system through which referrals are placed on a child and adolescent mental health services (CAMHS) pathway, or (ii) use a digital portal (myHealthE [18] or Interactive CAMHS Assessment Network) to screen for hyperactivity/impulsivity, inattention and challenging behaviour using the Strengths and Difficulties Questionnaire [19] and to establish consent for contact (C4C).

### Participants

Participants in the study will be parents of children aged 5 to 11 years that passed the initial triage and have been accepted onto the services’ assessment waiting list and the children’s teachers.

#### Inclusion criteria

- Parent of a new referral. A ‘new referral’ is defined as a child who has been on a waitlist for no longer than six calendar months.
- A routine positive screen for child’s hyperactivity/impulsivity, inattention and challenging behaviour using the SDQ (hyperactivity/inattentive subscale score ≥ 8 and conduct problems subscales score ≥ 4). These scores represent the “high” and “very high” problem groups according to the four-band classification of the SDQ cut off scores (top 10% of the population) [20].

#### Exclusion criteria

- Parent lacks access to a suitable electronic device: a smartphone with operating system OS 4.1 and later (Android devices) or iOS 9.0 (Apple devices).
- Child is living under Local Authority care.
- Parent has an insufficient level of English language.

Foster parents of children living under local authority care have been excluded, as many foster placements are shorter than 12 months, which will be the duration of the follow-up in the RCT of STEPS.

There will be no inclusion/exclusion for teachers, but researchers will need to obtain parents’ permission for contacting teachers.

### Intervention

Although this is not a study of the STEPS app, we provide a brief overview of the app for information (more details can be found in Supplementary Documents: 1. Structured E-Parenting Support). In STEPS, parents will be able to move through the content (steps) at their own pace and at any time of day. The order of the steps is fixed, but there will be a degree of choice within each step. The content will be delivered mainly using short videos and audio clips.

The STEPS app has one preparatory module, “Introduction”, followed by eight separate intervention modules (steps). How long STEPS takes to complete will depend on the pace and frequency of usage. However, each of the eight steps is designed to take about 20 min if completed in one go. Parents will be able to use STEPS for 12 weeks from the time they download the app. The access to the app will be blocked 12 weeks after download, as the team will not be able to monitor the participants after that time. There will be no restrictions on concomitant care or interventions.

### Procedure

**Fig. 1:**
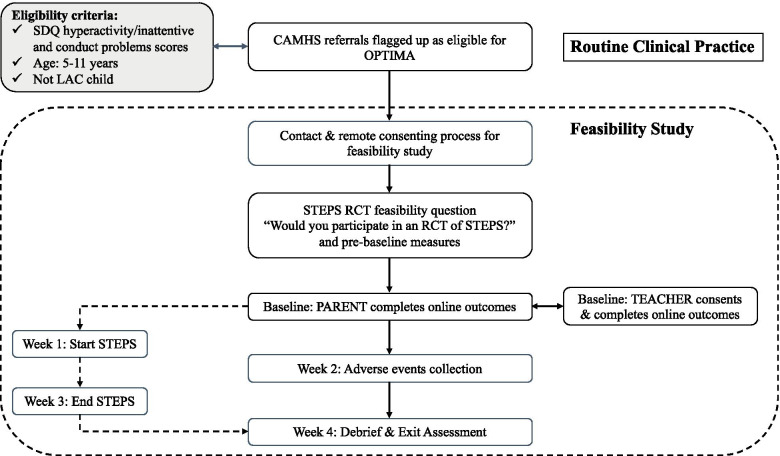
OPTIMA feasibility study flowchart. SDQ = Strengths and Difficulties Questionnaire; ADHD = attention-deficit/hyperactivity disorder; ODD = oppositional defiant disorder; LAC = looked after child; CAMHS = child and adolescent mental health services; OPTIMA = Online Parent Training for the Initial Management of ADHD referral

All study data will be collected remotely: online using Qualtrics, which is a well-established data collection platform, or on the phone by a trained researcher. For each participant, assessments will take place over the period of 4 weeks with measures taken shortly after consent (pre-baseline: sample characteristics measures and the feasibility question) and then at 1 week (baseline: questionnaires completed by parents, including economics measures), 2 weeks (adverse events collection) and 4 weeks (exit assessment) post-consent. Teachers will be contacted as soon as parents have provided their consent. Also, see Fig. 1.

### Outcomes

The primary outcome for this study is the number of eligible feasibility study participants who *agree in principle* to take part in a full RCT of STEPS each month.

Agreement in principle to take part in the upcoming STEPS RCT: feasibility study participants will each be given a balanced explanation of what an RCT involves (see 2. Feasibility Question Script in Supplementary Documents) and asked. “*if there was an RCT of STEPS would they be willing to take part in it*?” After being asked the question parents will be given a choice of a YES/NO answer. Parents, who answer ‘NO’, will be asked to provide a brief explanation for their decision. The researcher will note a parent’s answer and any comments on the data form.

Secondary feasibility outcomes concerning the secondary study objectives are shown in Table 1.

**Table 1:** Secondary feasibility outcomes

To pilot the remote consenting procedure, an online outcome and adverse events collection procedure.	To assess the feasibility of collecting teacher data remotely within the required timeframe.	To capture technical difficulties with completing online questionnaires.
*Remote consenting procedure:* • Mean time taken to complete remote consenting procedures. • Parents’ mean ratings of satisfaction with consenting procedures. • Content analysis of text box evaluations.	• Mean time to identify teachers. • The proportion of teachers’ questionnaires returned within 7 days of receiving a link to an online survey out of the number of teachers recruited	• Qualitative description of technical difficulties with accessing or completing online questionnaires.
*Online outcome collection:* • The proportion of participants completing outcome questionnaires within 7 days of receiving a link to an online survey out of the number of participants who are in the study. • Mean number of reminder emails about completing outcome questionnaires sent to parents by the research team. • Parents’ mean ratings of satisfaction with online data collection. • Content analysis of free text evaluations.		
*Online adverse events collection:* • The proportion of participants completing the adverse events questionnaire within 7 days of receiving a link to an online survey out of the number of participants who are in the study. • Mean number of reminder emails about completing adverse events questionnaire sent to parents by the research team.		

### Measures and procedure

Figure 1 shows the study flowchart.

#### Pre-baseline measures

These will be collected directly online using Qualtrics or on the phone and entered by the researcher into Qualtrics to characterise the sample and will consist of the following questionnaires (for participant timeline see Table 2):

**Table 2:**
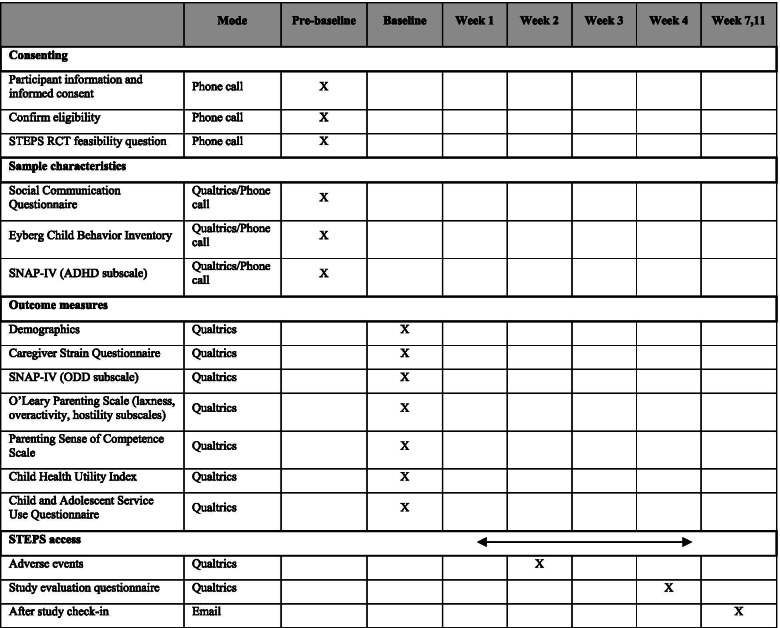
Feasibility study timeline (parents only)

Eyberg Child Behavior Inventory [21] is a 36-item measure of conduct problem behaviour for children aged 2–17 years. Parents rate behaviour occurrence on a 7-point scale ranging from never to always and the item ratings are summed to yield the Intensity score. Parents also indicate whether the behaviour is a problem at present by answering “yes”/“no”. The yes responses are summed to produce a Problem score.

Social Communication Questionnaire [22] is a 40-item screening measure for autism spectrum disorder (ASD). It is based on the Autism Diagnostic Interview-Revised [23] and validated to use for ages 4 years and above. Questions focus on behaviours that are likely to be observed by the primary caregiver and concern the following domains: reciprocal social interactions, language and communication and repetitive and stereotyped patterns of behaviours. The presence of autism-positive behaviour is coded as 1 and its absence as 0. Because the first question concerns the level of current language and as is not included in the total score, for children with language, the maximum score is 39; for children without language, the maximum score is 33 (language items are inapplicable). The recommended cut-off score for cases requiring further investigation is 15. Due to the high levels of clinical co-existence of ASD and ADHD [24], this questionnaire is used to describe how much ASD there is in this population.

ADHD subscale of the Swanson, Nolan and Pelham Rating Scale-the MTA version (SNAP-IV) [25]. The SNAP-IV is a behavioural rating scale that captures the core symptoms of ADHD and ODD. The ADHD subscale of SNAP-IV consists of 18 items that are rated on a 4-point scale (not at all, just a little, quite a bit, very much). The subscale scores are obtained by averaging responses regarding inattentive and hyperactive/impulsive symptoms. Scores above the 95th percentile are considered clinically significant. The questionnaire has established validity and reliability [26].

We will also obtain information about the child’s general practitioner’s name and address and a teacher’s name and the name of the school (if a parent consented to teacher contact).

#### Baseline measures—parents

At baseline, parents will answer demographic questions and complete clinical outcome and health economics measures online using Qualtrics (also see Table 2).

##### Demographics questionnaire

Information about the child’s age, gender, ethnicity, parental education, socio-economic status and family structure (specifically, about marital status).

##### Clinical outcome measures

The ODD subscale of the SNAP IV [25]. This subscale consists of eight items that are rated on a 4-point scale (not at all, just a little, quite a bit, very much). The subscale score is obtained by averaging responses. Scores above the 95th percentile are considered clinically significant. The questionnaire has established validity and reliability [26].

The O’Leary Parenting Scale [27] will be used to examine changes in parenting style. Parents will rate their probabilities of using specific discipline strategies in response to child misbehaviours. Responses are made on a 7-point scale. Only items that map onto the three factors: laxness (lack of consistent responding; 5 items), over-reactivity (overly emotional or harsh responding; 5 items), hostility (hostile verbal and physical behaviour towards the child; 3 items) will be included.

The Parental Sense of Competence scale [28] will be used to measure parenting satisfaction. The scale has 16 items and parents make responses on a 6-point scale, with options ranging from strongly disagree to strongly agree. Two indices are calculated for each participant: efficacy, which reflects domain-general parenting self-efficacy and satisfaction, which reflects the degree of anxiety, frustration and motivation associated with the parental role.

Caregiver Strain Questionnaire (CGSQ) [29]. CGSQ is a 21-item measure of self-reported strain experienced by caregivers and families of children with emotional and behavioural problems, with responses made on a 5-point Likert scale. It consists of three subscales: Objective Strain, Subjective Internalised Strain and Subjective Externalised Strain. Global Caregiver Strain Score is determined by calculating the mean of all items on the CGSQ.

##### Economic measures

Child and Adolescent Service Use Schedule (CA-SUS) [30], a measure of service use applied in a range of populations of young people with mental health problems. The CA-SUS will be completed by the primary carer at baseline (covering the previous three months). The CA-SUS collects information on the use of all hospital and community-based health services, social care and education services, service-provided accommodation (for example, Local Authority foster or residential care) and all prescribed medications.

Child Health Utility Index (CHU-9D) [31]. The CHU-9D is a paediatric preference-based quality of life measure for use in healthcare resource allocation decision-making. The CHU-9D has been designed for self-report by children aged 7 to 17, but with an interviewer’s help, can also be used in children as young as 6 years old [32] and guidance is available from the developers for proxy completion by parents for children aged 5 and under. The questionnaire includes 9 items, each with a 5-level response category. Each item taps into a different domain of children’s present functioning: worry, sadness, pain, tiredness, annoyance, school, sleep, daily routine and activities. The present study does not involve collecting data from children; therefore, a proxy version will be used for all.

#### Baseline measures—teachers

Teacher data will be collected online using Qualtrics and will consist of the ODD subscale of the SNAP-IV [25].

#### Adverse events

Parents will be asked to report any physical and mental health difficulties or events for themselves and their child that have occurred since the start of the study. This information will be collected online during week 2 to pilot the adverse event collection procedure (see Table 2). A more detailed description of adverse events recording and reporting is provided in ‘Adverse events management’.

#### Exit interview: evaluation of acceptability

At the end of week 4, parents will be emailed a link to a brief evaluation questionnaire (see Table 2). The questionnaire will aim to collect information about parents’ satisfaction with remote data collection and their feedback about study procedures. Responses will be provided using structured Likert-type scales and free text boxes.

### Participant timeline

Table 2 shows a detailed schedule concerning the study timeline for parents participating in the study.

### Sample size

**Fig. 2:**
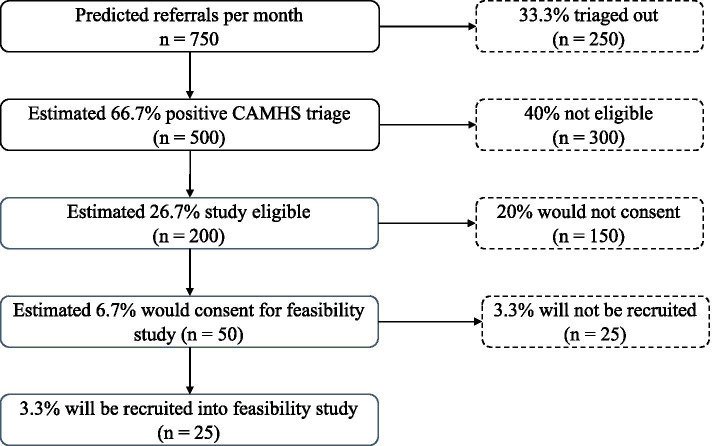
Estimated monthly referral flow

The primary aim of the feasibility study is to establish that we can recruit enough parents of new referrals for the STEPS RCT using our remote strategy incorporating existing routine CAMHS triage and clinical screening elements using MHE, ICAN or telephone-based methods across the five recruitment sites (Fig. 2). Based on the power calculations for the larger planned RCT of STEPS, we need to recruit 13 participants a month (352 families over 27 months). We will base our assessment of whether this is possible on the primary feasibility outcome described above, the rate per month of participants who answer the STEPS RCT feasibility question in the affirmative. Because we assume it is more likely that people would agree in principle to take part in STEPS RCT during the feasibility study than would actually consent to the real trial, we have adopted a conservative requirement in this feasibility of 18 (rather than 13) parents a month as our aim in this feasibility study and our stop/go criterion for the larger trial.

The numbers of potential participants at each stage of the selection process during each month of the feasibility study is shown in Fig. 2. Based on audit and best information across our trial sites, we estimate that each month, parents of 200 newly referred children (of roughly 750 referrals across the 5 sites) will be eligible to take part in the feasibility study. We estimate that 50 parents would consent to the feasibility study and of these roughly 25 will give a positive response to the feasibility question, which is above the number required.

We will test this by recruiting 25 study eligible parents (half of the number available) each month (100 over 4 months). Our projections will be supported if 12 or so of these parents answer the feasibility question positively. This number will also give us a good sample in which to assess our other feasibility measures.

### Recruitment

As part of our remote strategy, participants will be identified using one of three methods—depending on the current practice of the Trust from which they are being recruited:

#### Method 1: myHealthE portal

In some of the participating organisations, CAHMS triage and rapid screening for hyperactivity/impulsivity, inattention and challenging behaviour occur as part of routine care using myHealthE (MHE; https://www.slam.nhs.uk/our-services/camhs/get-involved/myhealthe/)—a Caldicott Guardian approved, General Data Protection Regulation (GDPR) compliant, online portal for the automated screening of referred families using NHS CAMHS data [18, 33]. MHE uses a secure text/email system through which primary caregivers are invited to register and complete validated clinical screening measures using an online portal. These are then automatically coded using standard algorithms to subsequently allow the research team to identify potentially eligible parents. MHE also seeks parents’ permission to be contacted with invitations to take part in research studies; that is, provide consent-for-contact. By consenting to research contact, parents also give their permission for Trust-approved researchers to review their children’s medical records to establish eligibility.

Authorised staff at each participating clinic site will regularly input the new referrals’ details into MHE, which will initiate the mechanism for obtaining the initial consent for research contact and for obtaining the screening information. Members of the research team will regularly log into the MHE researcher portal (this portal only includes information about children’s whose parents gave consent for contact) to check for any new cases flagged up as ‘eligible’. At each organisation, a member of the research team will identify potential participants by reviewing cases flagged up as ‘eligible’ by MHE using a standard algorithm. To be flagged as ‘eligible’ the case has to fulfil the following criteria: (1) a child is aged 5 years 0 months to 11 years 11 months, (2) a child screened positive for ADHD-type problems and challenging behaviour on the SDQ; (3) a child is not under local authority care; and (4) a parent provided routine consent for research contact. The ‘eligible’ flag will be treated as a potential participating family.

#### Method 2: Interactive CAMHS Assessment Network (ICAN)

In NELFT, routine outcome measures are collected through the Interactive CAMHS Assessment Network (ICAN). It is a digital application in which clinicians, young people, parents, carers and teachers can provide routine clinical measures. ICAN provides useful feedback to clinicians to help track progress and data in real-time and is directly linked to the electronic patient record.

Parents of patients, who provide consent for research contact through ICAN, will be asked to complete the SDQ also through ICAN. This information will be then passed onto the research team, who will review the SDQ scores to establish eligibility. Parents of children who meet age and the SDQ eligibility criteria will be approached by the researcher with an invitation to take part (by email, phone, or post).

#### Method 3: Manual review of current referrals

These methods can be adopted by any organisation participating in the study to either supplement digital identification (via MHE or ICAN) or as a sole way of identifying the eligible families.

- Consent for contact: members of the clinical teams ask parents of patients if they are happy to be contacted about any relevant research. If they answer yes, this is recorded in their electronic health record and gives Trust-employed Clinical Study Officers (CSOs) or researchers permission to screen their record for suitability and then make direct contact with the parent to invite them to the study.
- Clinic referral: a member of a clinical team will specifically discuss the study with the parent during a routine clinical contact and if they are interested and give permission then pass their details to CSOs or researchers to take forward from there. This will act as their referral to the study. Information about the study, including a link to access the online information sheet, may also be emailed or posted to families.

### Data collection

Two categories of data will be collected in the feasibility study: (1) participants’ reports on the outcome measures and information about adverse events and (2) the STEPS app usage data (if a participant chooses to use the app).

1. Outcome data and information about adverse events will be collected online using Qualtrics—a secure dedicated survey platform and over the phone by a trained researcher.

#### Online data collection

Researchers will email participants a link to the relevant questionnaires. Participants will need to authenticate (log in) to the online platform using a password before they can view and complete the outcomes survey. After the exit assessment of the final participant is completed, data will be exported and entered into the study database.

#### Phone data collection (pre-baseline)

A trained researcher will ask the feasibility question and administer the three questionnaires during a phone call with a parent. Responses will be logged by a researcher directly into Qualtrics. If requested, parents will be able to complete the three questionnaires online in their own time.

(2) All usage data will be collected by Firebase Analytics and will be stored with Google servers. Usage data held within Google Firebase will be generally of non-identifiable nature. Therefore, the risk of harm to participants following a potential privacy breach is low. After data collection has been completed, data will be transferred into the Excel database and Google Firebase account and all the data contained within the account will be deleted.

### Data management

All feasibility, clinical and economic measures will be stored in Qualtrics IBM-SPSS exports and/or a study IBM-SPSS database. The app usage data for the feasibility study will be stored in an Excel database. These databases will be stored on a secure KCL network drive, accessible to the study team only. The study team will provide data extracts to the trial statisticians, upon request.

Data validation will be carried out at the time of data import or entry into the respective SPSS databases to ensure that entered values are valid (e.g., for dates, within an allowed period; for continuous variables, within an allowed range; for categorical, within a list of allowed responses). These validity criteria will be specified in a database specification document, stored in the trial master file.

The study SPSS databases will be stored in a version control system, such that changes made over time can be examined and recovered. All databases will be registered in the King’s Data Protection Register (KDPR).

Any potential data protection breach incidents will be managed in line with KCL Data Breach Management Procedure. For details of this procedure see: https://www.kcl.ac.uk/governancezone/assets/governancelegal/data-breach-procedure.pdf.

De-identified data and identifying information will be stored for seven years from the completion of data collection. After this time, any identifying information will be securely destroyed, and fully anonymised data will be transferred to King’s College Research Data Management system for long-term storage.

### Statistical analysis

This feasibility study will not use inferential statistics to assess the primary objective but will estimate the rate per month of eligible participants who agree in principle to take part in the upcoming STEPS RCT (with the aim of getting 18 per month to give sufficient statistical power to evaluate STEPS in the larger trial planned for later in the programme). Other objectives will be met by using descriptive statistics and qualitative assessments in combination to study the efficiency and acceptability of aspects of the proposed methods. Neither subgroup nor interim analyses are planned in this study. A quantitative statistical analysis plan is being developed.

### Data monitoring

The Programme Steering Committee (PSC), a body independent of the research team, will provide formal oversight and expert advice for the overall OPTIMA programme, which includes oversight over the present feasibility study. Its role is to ensure that the trial is conducted in a rigorous and timely manner and consider any proposed changes to the agreed programme of research. Initially, the PSC will meet every six months with one meeting coinciding with the end of the feasibility study, to determine whether the project should progress to the RCT. The PSC consists of an independent chair, statistician, digital health expert, digital mental health interventions expert, two parents of a child with ADHD, and a health economics expert. Recruitment to the study will be rapid and no interim analyses are planned so a separate Data Monitoring and Ethics Committee (DMEC) will not be formed. However, we reserve the option to form one if the PSC deem it necessary at any point during the study.

### Adverse events management

All adverse events (AE) will be recorded on the OPTIMA Adverse Events Form. The record will include a brief description of the event, any clinical symptoms and a date when the event started and stopped, and who was involved (e.g., a child, a parent, another family member). The researchers will be recording any AE they noted in their communications with the participant at any point during the study. In addition, to adverse events disclosed spontaneously by participants, information about adverse events will be collected online at 2 weeks post-baseline assessment using OPTIMA adverse events questionnaire.

All completed adverse event forms will be reviewed by the research team to identify any Suspected Unexpected Serious Adverse Reaction (SUSAR). If an event meets the definition of a SUSAR (any serious adverse event that is related to the trial intervention and unexpected, i.e., not listed in the protocol as an expected side effect of the intervention) then the member of staff that received the information must inform the Chief Investigator (CI) within 24 h of becoming aware of the serious adverse event and provide all necessary information. Each case will be assessed by the CI, team clinical expert and medical expert for seriousness, expectedness and relatedness to the study. The CI will then take appropriate action, which may include halting the study and informing the Sponsor of such action. Events categorised in the protocol as an expected (see Table 3), do not need to be reported.

**Table 3:** Expected adverse events

School & community	Family dynamics	Parental wellbeing
• Increased child refusal to go to school/community activities. • Exclusion from school/community activities.	• Deterioration in child behaviour (including self-harm) or wellbeing. • Deterioration in sibling wellbeing (including self-harm). • Increased family discord. • Breakdown in family structure. • Social work involvement or child protection concerns.	• Increased depressed mood • Increased anxiety/stress • Increased tiredness/fatigue • Increased/decreased sleep

STEPS is a psychological intervention, not pharmacological, and so physical adverse reactions are not expected. Medical adverse events will be recorded using standard medical definitions.

### Ethics

The application for ethical approval for the study was submitted to the London-Riverside Research Ethics Committee (REC) and received a favourable opinion on further information on 17 November 2020, REC reference number: 20/LO/1173.

To allow maximum flexibility in terms of recruitment, a substantial amendment to the ethical application was submitted to the REC in March 2021. This amendment introduced two additional methods of participant identification (Method 2: ICAN and Method 3: a manual review of referral lists). This amendment was approved in April 2021. The REC was notified of two further non-substantial amendments, which did not require study-wide review, one in May 2021 and the second one in June 2021. The current manuscript is based on protocol version 5.0 dated 10 June 2021.

Recruitment for the study commenced in April 2021 and will continue until the end of July 2021.

### Protocol amendments

In case of new information becoming available, which may result in significant changes to the risks and benefits of taking part, the Participant Information Sheet and informed consent form will be reviewed and updated accordingly. All participants actively enrolled in the study will be informed of the updated information and will be given a revised copy of the Participant Information Sheet and informed consent form to confirm their wish to continue taking part.

### Consent

#### Parents

All participants will provide written informed consent to take part in the feasibility study. The informed consent form will be provided in electronic form using Qualtrics to be completed by the participant before they enter the study. The researcher will explain the details of the study, provide the Participant Information Sheet and will answer any questions that the participant has concerning the study. They will also inform parents that participation is completely voluntary, and they are under no obligation to take part and they can withdraw at any time without their care being affected. The researcher will also tactfully check whether it is a convenient time for a parent to commit to the study (i.e., if there is no expected imminent change to their existing care commitments, for example, late stage of pregnancy). On the consent form, there will be a separate statement requesting permission to contact the child’s teacher. If this box is not ticked, the teacher will not be contacted. If parents are unable to understand the consenting process it will be assumed that they do not meet the language requirement for the study.

#### Teachers

Parents, who have given consent to contact their child’s teacher, will be asked to provide the name of their child’s school and the teacher’s name. The research team will then contact the teacher (by phoning or emailing a school) and invite them to take part in the study. An invitation will be followed up with an email containing a link to the teacher survey. This survey will include Teacher Information Sheet, a consent statement and a questionnaire. Teachers will also be invited to contact the research team with any questions that they might have about the study before they agree to take part.

### Confidentiality

The CI and all members of the research team will take every effort to preserve the confidentiality of participants taking part in the study. To de-identify the data, each participant will be assigned a study ID. Participants identifiable data required for administrative purposes (e.g., name and contact details) will be stored in a separate, encrypted, password-protected file. These will be accessed only by those members of the research team who are responsible for contacting participants (e.g., to email a link to the online survey). No individual participant’s data will be identifiable in the publications or reports that may result from this study.

### Post-study care

Participants will continue to have access to the STEPS app for 9 weeks after the study ends. This means that overall, participants will have access to the intervention for 12 weeks.

During this time, the researchers will check in with each participant every 4 weeks (by email) and provide contact details for the local clinical services in case a parent/caregiver has any major concerns about themselves or their child. The check-in email will also remind the participant when their app access is due to expire.

## Discussion

The long waiting lists to receive assessment and treatment from children’s mental health and paediatric or early behavioural help services mean that parents can be left without support and guidance at the time when it is most needed. Furthermore, anecdotal evidence suggests that due to the COVID-19 pandemic-related disruptions, families wait for support longer than ever before. Given that over 80% of adults in the UK own a smartphone, the use of a digital application to deliver support and training to parents on the waiting list could provide a clinically effective and low-cost solution to support families when in-person clinical support is not available. The planned RCT will seek evidence whether the STEPS app is a clinically and cost-effective intervention for parents of hyperactivity/impulsivity and inattention children with challenging behaviour during the time they remain on the waiting list for assessment.

However, recruiting from the waiting list is challenging. First, the traditional methods, for example, clinician referral, are not feasible due to the very limited contact between the clinician and the family during that time. Second, obtaining consent for research contact that would allow the researchers to get in touch with the family and invite them to participate in the study is also limited, as this is typically discussed during the assessment visit. Moreover, while the number of health research studies that adapted to remote consenting and data collection procedures has rapidly increased in recent months due to the COVID-19 pandemic-related restrictions on in-person contact, the evidence showing that participation in remote health studies is acceptable is lacking. This is especially important in the context of conducting research with parents, who are often more difficult to reach and engage due to a complex set of challenges in the families (e.g., parental ADHD or mental health concerns).

To ensure the timely and efficient evaluation of STEPS in OPTIMA, we have worked with children’s services to implement a remote strategy for recruitment, screening and assessment of recently referred families—part of which is incorporated into routine clinical practice and part of which is OPTIMA specific. The purpose of this study is to establish whether we can use this approach to recruit an adequate number of participants to run a randomised controlled trial and to measure whether obtaining consent and collecting data from parents and teachers using remote methods (online and on the phone) is acceptable to the study participants. We will use three different methods of identifying participants: this will include the new digital platforms that allow to obtain screening information and collect consent for research contact, as well as traditional manual methods of recruitment. This will maximise the opportunities for recruitment by involving a wide range of health services. Although the originally approved study protocol included only one option of identifying participants (through MHE; see Method 1: myHealthE portal), our discussions with the services that were interested in joining as recruitment sites revealed that it was not always possible to adopt digital platforms. In response to this, a substantial protocol amendment was submitted for ethical review to allow other methods of participant identification. It is also possible that other sites may not have the capacity to support manual recruitment and adopting digital methods may be the only viable option. Adopting a range of approaches to identifying participants, will also allow us to establish which of the methods is most effective when recruiting from the waiting list and inform the approach. Therefore, the results from this study may provide valuable information for other researchers planning to recruit a substantial number of participants from the waiting list.

Finally, for practical as well as scientific reasons, the present study, as well as the future RCT of STEPS, will necessarily have to focus on parents with an adequate level of English who have access to a smartphone. However, it is important to acknowledge that these eligibility criteria may reduce participation opportunities for parents from ethnic minorities or from more deprived areas. We plan to work with OPTIMA Patient and Public Involvement Panel, as well as with the newly launched NIHR Maudsley Biomedical Research Centre Race and Ethnicity Advisory group on identifying feasible solutions that could help reduce the barriers for inclusion.

### Study status

At the time of manuscript submission, the study was open to recruitment. Recruitment has now been completed.

## Supplementary Information

**Additional file 1.** Structured E-Parenting Support (STEPS).

**Additional file 2.** Feasibility question script.

## Data Availability

Not applicable.

## Abbreviations

ADHD: Attention-deficit/hyperactivity disorder
AE: Adverse event
CAMHS: Child & Adolescent Mental Health Services
CI: Chief investigator
DMC: Data Monitoring Committee
ICAN: Interactive CAMHS Assessment Network
ICF: Informed consent form
MHE: MyHealthE
ODD: Oppositional defiant disorder
RCT: Randomised controlled trial
REC: Research Ethics Committee
SUSAR: Suspected Unexpected Serious Adverse Reaction
SDQ: Strengths and Difficulties Questionnaire
STEPS: Structured E-Parenting Support
PSC: Programme Steering Committee
