# Supplementary material for: The feasibility of a strategy for the remote recruitment, consenting and assessment of recent referrals: a protocol for phase 1 of the On-Line Parent Training for the Initial Management of ADHD referrals (OPTIMA)
Source: Pilot Feasibility Stud. 2022 Jan 3;8:1. doi: 10.1186/s40814-021-00959-0 (PMC8720938; doi:10.1186/s40814-021-00959-0)
Supplement: Supplementary file 2 — Additional file 2. Feasibility question script. [file 40814_2021_959_MOESM2_ESM.pdf]

Supplementary document 2: Feasibility question script

Thank you for agreeing to take part in our study.

Eventually, we would like to let everyone have access to STEPS. But, before we can do that, we need to show the Government that it works. The only way to be sure of that is to run what is called a randomised controlled trial (or RCT for short). I will explain what an RCT is and how it would be different from the study we are conducting now.

First, in an RCT, only half the parents taking part would receive the app for 10 weeks and the other half remain on the waiting list as usual. You would not choose which group you are in – that will be done at random by a computer. So, you would have a 50:50 chance of getting the STEPS app.

Second, the RCT study would be longer than the current study. You would be involved with it for a year. During that that time we will contact you to fill in online questionnaires at regular intervals. So, you would need to be willing to be contacted by the research team several times across the period of one year.

In summary, this RCT involves:

- Taking part in the study for 12 months rather than 2 and completing more questionnaires
- Having a 50% chance of receiving the STEPS app.

We are asking parents like you, whether if you were asked to take part in an RCT of STEPS you would agree.

Now I have explained the STEPS RCT to you, its importance and what it would involve, what would your answer be?

☐ YES, I would be willing to take part in the RCT of STEPS.

☐ NO, I would not be willing to take part in the RCT of STEPS.

If your answer is NO, please help us by providing a reason why you would not want to take part:

____
